# Changes in taxonomic and functional diversity of fish communities after catastrophic habitat alteration caused by construction of Three Gorges Dam

**DOI:** 10.1002/ece3.6320

**Published:** 2020-04-28

**Authors:** Chen Zhang, Masami Fujiwara, Michaela Pawluk, Huanzhang Liu, Wenxuan Cao, Xin Gao

**Affiliations:** ^1^ Key Laboratory of Aquatic Biodiversity and Conservation of Chinese Academy of Sciences Institute of Hydrobiology Chinese Academy of Sciences Wuhan China; ^2^ University of Chinese Academy of Sciences Beijing China; ^3^ Department of Ecology and Conservation Biology Texas A&M University College Station TX USA; ^4^ Department of Wildlife and Fisheries Sciences Texas A&M University College Station TX USA

**Keywords:** assembly, diversity–disturbance relationship, habitat alternation, regime shift, Three Gorges Dam

## Abstract

Habitat alterations that result from anthropogenic disturbance impact both the abiotic and biotic conditions of ecosystems, causing changes in biodiversity in many parts of the world. Recently, the use of functional diversity has been suggested as an approach to better evaluate the effects of such disturbance on particular communities. Here, we investigated the temporal changes in species and functional diversities of fish communities in the downstream area of the Three Gorges Dam (TGD) before, during, and after impoundment. We found two regime shifts in the fish community in 2004 and 2013 following impoundment. Although taxonomic diversity declined sharply at the first regime shift, it increased at the second shift. On the other hand, functional diversity declined throughout the same period, indicating the loss of functional diversity despite increased species diversity. Our analysis also showed that the fish communities shifted from under‐dispersion to over‐dispersion due to both a decrease in the relative abundance of migratory fish and an increase in the number of fish adapted to the new hydrologic conditions. Our results indicated that the impacts of dams on downstream fish communities may change over time. Interactions between species may become more important when the environment is stable.

## Graphical abstract

We investigated the temporal changes in taxonomic and functional diversities of fish communities in the downstream area of the Three Gorges Dam (TGD).We found two regime shifts in the fish community and the opposite trends in taxonomic and functional diversities.

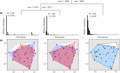

## 1 INTRODUCTION

Habitat alterations driven by humans are major causes of changes in species biodiversity and community composition (Burlakova et al., 2011; Forister et al., 2010; Tilman, May, Lehman, & Nowak, 1994). Aquatic ecosystems are particularly vulnerable to habitat alterations and disturbance (Dudgeon et al., 2006; Pereira et al., 2010; Winemiller, 2018), and aquatic habitats have changed dramatically in many rivers worldwide, especially due to dam construction (Ligon, Dietrich, & Trush, 1995; Zarfl, Lumsdon, Berlekamp, Tydecks, & Tockner, 2015). The ecological impact of dams on fishes is well documented (Kingsford, 2000; Liermann, Nilsson, Robertson, & Ng, 2012; Poff, Olden, Merritt, & Pepin, 2007). In recent decades, the impact of hydroelectric dams on the functional composition of fishes has attracted more attention (Arantes, Fitzgerald, Hoeinghaus, & Winemiller, 2019; Fitzgerald et al., 2018).

A functional trait‐based approach can better quantify the impacts of dams on ecological communities because ecosystem processes are more directly linked to functional diversity than to species diversity (dos Santos et al., 2017; Lima et al., 2018; Pool, Olden, Whittier, & Paukert, 2010). This is especially true for fishes, for which functional traits such as morphological characteristics (Langerhans, Layman, Langerhans, & Dewitt, 2003; Lujan, German, & Winemiller, 2011; Schlosser, 1982), life‐history strategies (Fujiwara, 2007; Mims & Olden, 2012; Olden, Poff, & Bestgen, 2006), and feeding mode (Troia & Gido, 2015; Winemiller, Fitzgerald, Bower, & Pianka, 2015) are closely associated with habitat conditions and food resources. Due to the close relationship between traits of fish communities and habitat alteration, the impact of dams on fish functional composition can be predicted (Arantes et al., 2019).

Species diversity and functional diversity are closely associated (Alahuhta et al., 2019; Cadotte, Carscadden, & Mirotchnick, 2011). However, they are sometimes complementary to each other, and at other times, they may have a strong positive correlation due to strong disturbance (Bihn, Gebauer, & Brandl, 2010; Heino, 2008). For example, in aquatic ecosystems, habitat alterations increase the risk of invasions by non‐native species and the promoting of the number of generalist species (Barthem, Brito Ribeiro, & Petrere, 1991; Forsberg et al., 2017), which may increase taxonomic diversity for a short period of time (Villéger, Miranda, Hernández, & Mouillot, 2010). However, in the long‐term, habitat alterations can aggravate the decline of specialist species and biotic homogenization (Mims & Olden, 2012; Pool et al., 2010), decreasing the functional diversity of fish communities (Cheng et al., 2014; Mouillot et al., 2014). Therefore, a key to understanding the effects of dam on ecological communities is to account for both species and functional diversities (Lima, Sayanda, Soares, Wrona, & Monaghan, 2017; Perônico, Agostinho, Fernandes, & Pelicice, 2020).

The Yangtze River is one of the largest rivers in the world, serving as a habitat for nearly 400 species or subspecies of fishes (Fu, Wu, Chen, Wu, & Lei, 2003). The Three Gorges Dam, the largest hydroelectric dam in the world, was constructed at the upper section of the middle reach of the Yangtze River. This section of the river was regarded as a biodiversity hotspot (Myers, Mittermeier, Mittermeier, Fonseca, & Kent, 2000; Olson & Dinerstein, 1998). The Three Gorges Reservoir (TGR), located immediately above TGD, was filled in three stages. The first stage raised the water level to 135 m above sea level (ASL) in 2003, and the second stage raised the level to 156 m ASL in 2006. The reservoir was filled to 172.5 m ASL in 2008 and then to 175 m ASL in 2010. Since then, the water level is reduced to 145 m from May to September, and it is raised to 175 m in the other seasons (Gao et al., 2019). TGD caused dramatic changes in the ecosystem by fragmenting the habitat and altering flow amount and pattern. These physical changes were predicted to impact species composition and biodiversity in the region (Wu, Huang, Han, Xie, & Gao, 2003). Changes in the environment downstream of TGD have been documented (Fu et al., 2010; Li, Dong, et al., 2013; Li, Xiong, Xiong, Dong, & Zhang, 2013), and they are known to have affected some important commercial and rare fishes and their habitats (Gao, Lin, Li, Duan, & Liu, 2014, 2016; Yi, Wang, & Yang, 2010).

In this study, we investigate the changes in species and functional diversities of fish communities downstream of TGD caused by the impoundment. The time series of fish abundance for 57 species obtained from the monitoring conducted from 1999 to 2015 with known functional traits gave us a unique opportunity to investigate changes in species and functional diversities, and compositions before, during, and after the series of TGD and TGR constructions. Our results contribute to understanding the process whereby large dams change fish assemblage and how the new species composition is shaped after a major habitat disturbance.

## 2 METHODS

Daily flow data in Yichang reach were obtained from the hydrologic information website of the Three Gorges corporation of China. The time series were from 1 January 1999 to 31 December 2015. Maximum discharge, minimum discharge, and mean discharge of each year were interploated by a local polynomial regression smoother (Cleveland, Grosse, & Shyu, 2017). The trends of maximum discharge, minimum discharge, and mean discharge were tested using Mann–Kendall test (Hamed & Rao, 1998).

**FIGURE 1:**
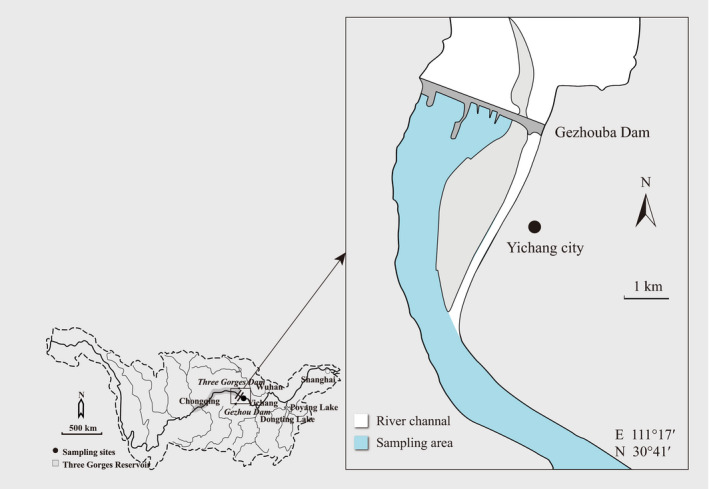
Map of downstream of Gezhouba Dam and fish survey area

Fish were sampled in the Yichang reach immediately downstream of the Gezhouba Dam (GZD; 30°43′N, 111°15′E; Figure 1) in the Yangtze River. GZD is located 38 km downstream of TGD. Sampling was done for approximately 20 days between October 20 and November 30 in each year from 1999 to 2015, over an approximately 20 km long stretch of the river. Fish were collected by local fishers each day using drift gill nets with mesh size ranging from 2 to 6 cm (30–40 m long × 1.0–1.5 m high). We selected 1–4 local fishing boats for each season, and they were surveyed almost every day during a sampling period. In most cases, fishing nets were set around 3:30 a.m., and fish were collected around 7:30 a.m. after nets drifted with the current for 4 hr. Mean catch per unit effort was 71 individuals per day. Each specimen was identified, counted, measured for body length (to the nearest mm), and weighed (to the nearest g). In the present study, we focus on fishes in midstream rather than riparian; thus, drift gill nets were determined to be the most appropriate fishing gear. As is the case with any fishing gear, a drift gill net has its inherent bias (e.g., size selectivity); however, it gives consistent samples of fish community across time (Gao, Zeng, Wang, & Liu, 2010; Gido, Matthews, & Wolfinbarger, 2000).

For analysis of functional diversity, twelve fish traits were determined for each species according to habitat use, life‐history, and trophic groups, including seven continuous and five categorical variables (Appendix S1: Table S1). Traits values/categories were either extracted from the existing literature (Ding, 1994; Institute of Hydrobiology, 1976). When information was not available for a specific species, the mean value within the same or a similar genus was used (Liu & Wang, 2018).

Species diversity and species evenness of the fish community were measured by Shannon–Wiener diversity (*H*) and Pielou evenness (*J*), respectively. The two indices were calculated as follows:

$$H=-\sum_{\left(i=1\right)}^{S}p_{i}\ln\left(p_{i}\right)\;\text{and}\;J=H/\log_{2}\left(S\right),$$

where *S* is species richness, and *p_i_* are species relative abundances (Hill, 1973). To further characterize a fish community, five functional diversity indices were calculated: functional richness (FRic), functional evenness (FEve), functional divergence (FDiv), Rao's quadratic entropy index (RaoQ), and functional redundancy. Functional richness (how much functional space is filled by the community), functional evenness (how regular species relative abundance is distributed in the functional space), functional divergence (how species abundances diverge from the center of the functional space), and Rao's quadratic entropy (how the sum of pairwise distances between species change) were calculated based on a multifaceted framework (Villéger, Mason, & Mouillot, 2008). Functional redundancy (i.e., where species perform similar roles in communities) was calculated following the procedure described by Ricotta et al. (2016). We also calculated community‐weighted mean trait values (CWM) to analyze the relationships between species composition and environmental conditions (Ricotta & Moretti, 2011).

For detecting shifts in fish community structure, we used the multivariate regression tree (MRT) and the sequential *t* test analysis of regime‐shifts algorithm (STARS) (Rodionov, 2004, 2006). MRT is a chronological clustering method, and each cluster represents a species community (De'ath, 2002). Combined with MRT, STARS was used to identify significant shifts in community time series data (Rodionov, 2004). Fish community structures were first analyzed with principal component analysis (PCA) applied to the matrix of species relative abundance. Before PCA analysis, the fish data were Hellinger standardized to reduce the influence of abundant species. The first and second principal component axes (PC1 and PC2) representing major changes of the fish community were passed through a white‐noise filter using the ordinary least‐squares methods (Rodionov, 2006) before the subsequent STARS analysis. We set the significance level at 0.05 and the window size at 3 or 5. We also used MRT with a species abundance matrix to find the existence and time point of a shift in community composition.

Based on the detected regimes, we separated data into time segments, each representing a regime. Then, we calculated Shannon–Wiener diversity and Pielou evenness for each regime. The similarity percentage (SIMPER) procedure was used to identify species that were most responsible for the Bray–Curtis dissimilarity between each regime (Clarke, 1993). Functional diversity indices were calculated using Gower's distance because we had both continuous and categorical trait variables (Pavoine, Vallet, Dufour, Gachet, & Daniel, 2009). Based on functional turnover and a functional nestedness‐resultant, the dissimilarity in functional composition of fish communities in each regime was calculated (Villéger, Grenouillet, & Brosse, 2013). Mean relative abundance of categorical trait groups was calculated, and the CWM values of longevity, age of sexual maturity, and fecundity were calculated for each regime. To remove the influence of species richness, functional diversity indices and CWM were compared with the null model results to control for differences in species richness (Mouchet, Villéger, Mason, & Mouillot, 2010). The null model used the matrix‐swap algorithm of Gotelli (2000); this method randomizes species occurrence frequencies by sampling. Simulations were run 999 times, and pairwise comparison of index differences between regimes was performed with one‐way permutational ANOVA (Ricotta et al., 2016). Standard effect size (SES) was calculated for each functional index and each regime as:

$$\left(mean_{\mathrm{observed}}-mean_{\mathrm{simulated}}\right)/SD_{\mathrm{simulated}},$$

where *mean_observed_* was the mean observed index, and *mean_simulated_* and *SD_simulated_* were the mean and standard deviation of simulated null model indices, respectively. Negative SES values indicated trait convergence, while positive values indicated trait divergence (Götzenberger et al., 2012). The significance of the difference from null expectations was tested using a one‐tailed test (*p* < .05) proposed by Swenson (2014). STARS were performed using Excel VBA (Rodionov, 2004, 2006). The other statistical analyses were performed using R software (R Core Team, 2017). Taxonomic diversity indices and SIMPER results were calculated with the “vegan” package, functional diversity indices and community‐weighted mean trait values were calculated with the “FD” package, and matrix‐swap null model and one‐way permutational ANOVA were performed with the “picante” package and “rcompanion” package, respectively.

## 3 RESULTS

**FIGURE 2:**
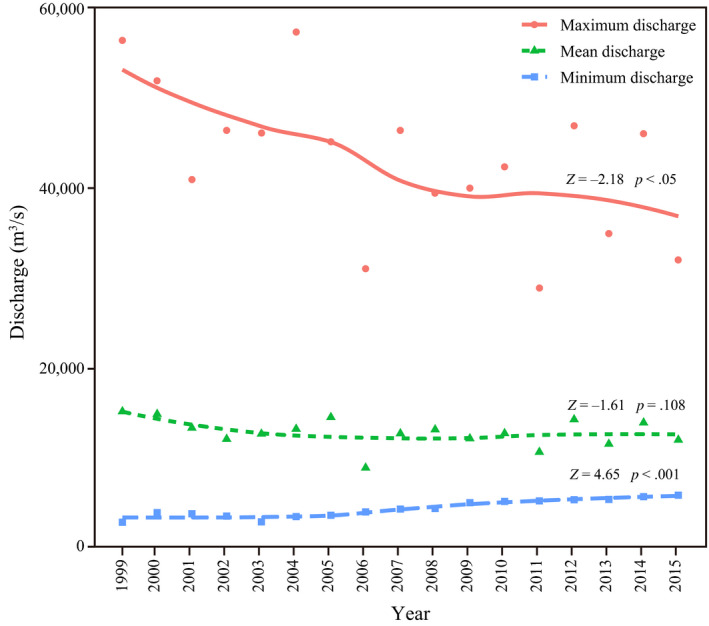
Maximum discharge, minimum discharge, and mean discharge in Yichang reach are plotted against year. Test statistics refer to Mann–Kendall test. Negative/positive *Z* values represent decreasing/increasing trend of data series

Results of Mann–Kendall test showed that maximum discharge in Yichang reach decreased while minimum discharge increased over time (Figure 2). Mean discharge did not showed significantly decreasing trend.

A total of 26,565 individuals representing 57 species in 9 family were caught from 1999 to 2015. Cyprinidae and Bagridae species accounted for 87% and 11%, respectively, of the total abundance. The fish communities were dominated by *Coreius heterokon* (43%), *Coreius guichenoti* (25%), and *Pelteobagrus vachelli* (8%) based on the cumulative abundance throughout the investigation.

**FIGURE 3:**
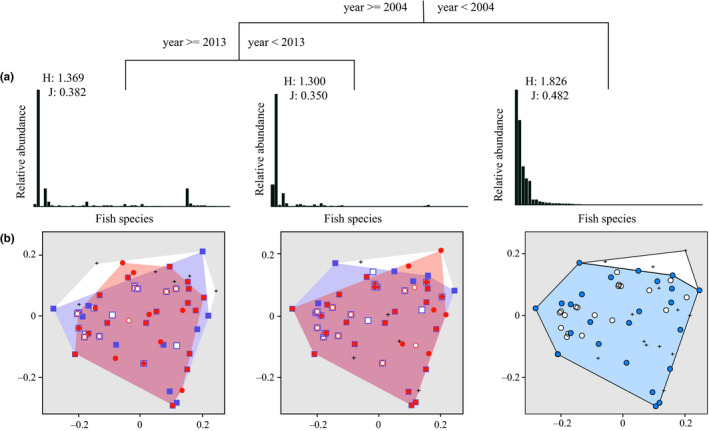
Chronological clustering by MRT and three successive structures of the fish communities. Fish species are ordered according to their relative abundance before 2004. *H* and *J* are values of Shannon–Wiener diversity and Pielou evenness, respectively (a). Functional spaces were plotted based on convex hull volume of functional richness in each regime. Functional spaces were regime 1, regime 1 compare to regime 2, and regime 2 compare to regime 3, from right to left (b)

The first and second principal components (PC1 and PC2) explained 62.17% of the variation in the fish community and were used in STARS for the detection of regime shifts. The results of STARS with both window sizes (3 and 5) indicated that the fish community could be separated into three regimes, with the change points identified in years 2004 and 2013 (*p* < .05) (Figure S1). The partitioning procedure implemented by MRT analysis produced a two‐split tree, explaining 65.6% of the community structure variance through the study period (Figure 3). The first split isolated 5 years before 2004, and the second split separated two periods (*n* = 9 and 3, respectively) as the cutoff point in 2013. Overall, results from both STARS and MRT consistently indicated that the fish communities in Yichang reach could be divided into three regimes: 1999–2003 (regime 1), 2004–2012 (regime 2), and 2013–2015 (regime 3).

Shannon–Wiener diversity and Pielou evenness of each regime (Table 1) were the highest in regime 1 and the lowest in regime 2. The two indices in regime 3 were higher than those in regime 2. The least number of species was observed during regime 3. Species evenness overall was low throughout the investigation, indicating that fish communities were dominated by several species. Compared with regime 1, some species adapting to lotic flow were not present in regime 2 (e.g., *Glyptothorax sinense*), but some eurytopic and limnophilic fish appeared (e.g., *Protosalanx hyalocranius*). Functional space during regime 3 was smallest and showed a tighter distribution around the functional center. Some species at the edge of the functional space disappeared, such as *Lepturichthys fimbriata* (Figure 3b).

**TABLE 1:** Median for FRic, FEve, FDiv, RaoQ, and FR for the three successional regimes

	Regime 1	Regime 2	Regime 3
FRic	0.003 (a)	0.002 (b)	0.002 (c)
FEve	0.403 (c)	0.467 (a)	0.420 (b)
FDiv	0.916 (a)	0.854 (b)	0.815 (c)
RaoQ	0.023 (a)	0.018 (b)	0.016 (c)
FR	0.786 (a)	0.770(b)	0.760 (c)

Note

Pairwise comparison of index differences between the successional regimes was performed with one‐way permutational ANOVA (*p* < .05). For each index, numbers followed by the same letter do not differ significantly.

Abbreviations: FDiv, functional divergence; FEve, functional evenness; FR, functional redundancy; FRic, functional richness; H, Shannon–Wiener; J, Pielou evenness; RaoQ, Rao's quadratic entropy.

Bray–Curtis dissimilarity indices between regimes were relatively high, indicating that fish community composition varied substantially during the study. SIMPER analysis showed that the difference between regime 1 and regime 2 was mainly caused by the increase of *Coreius heterodon* and the decrease of *C. guichenoti*, *Rhinogobio ventralis*, and *Rhinogobio cylindricus*, which together contributed 77.3% of the dissimilarity (Appendix S2: Table S1). Between regime 2 and regime 3, the decrease in the relative abundance of *C. heterodon* and *C. guichenoti—*as well as the increase in the relative abundance of *Siniperca chuatsi*, *P. vachelli*, and *Pseudolaubuca sinensis—*contributed a cumulative 62.4% of the dissimilarity (Appendix S2: Table S2).

Functional changes in community structure were analyzed with functional diversity and functional redundancy. The results of one‐way permutational ANOVA showed a significant difference in each functional diversity index between regimes (Table 1). FRic, FDiv, and RaoQ showed decreasing trends along with the three regimes, indicating the loss of several functional traits and change in the abundance of species with extreme functional traits (i.e., far from the center of functional space). For example, after the first filling of TGD, small migratory fish such as *Xenophysogobio boulengeri* and *Hemimzon sinensis* were absent in regime 2 and regime 3. Large migratory species such as *C. guichenoti*, *R. ventralis*, and *R. cylindricus* decreased sharply in their abundance. Higher functional evenness was found in regime 2 and regime 3.

**FIGURE 4:**
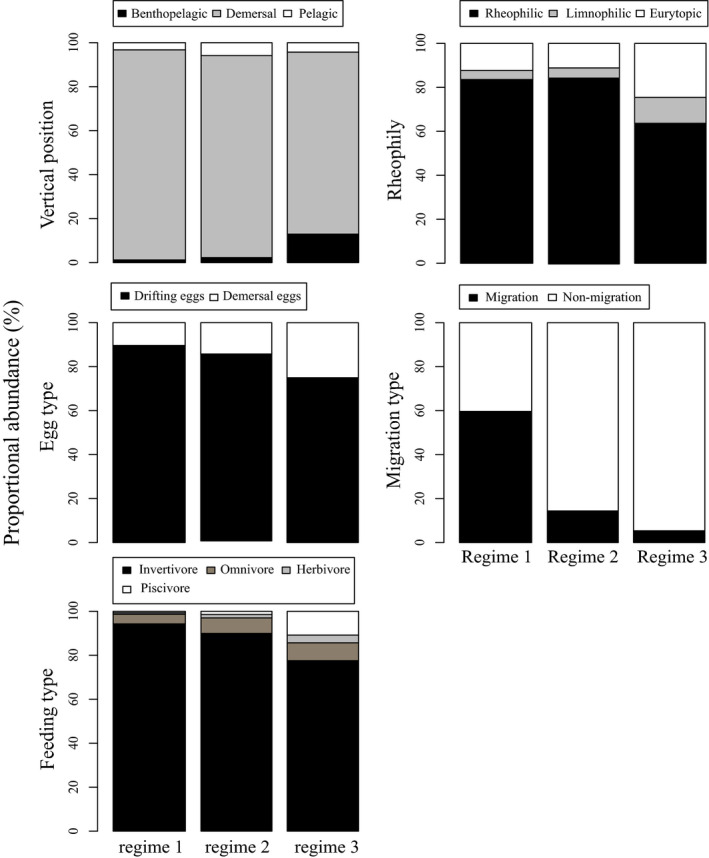
Mean relative abundance of functional groups in each regime

**FIGURE 5:**
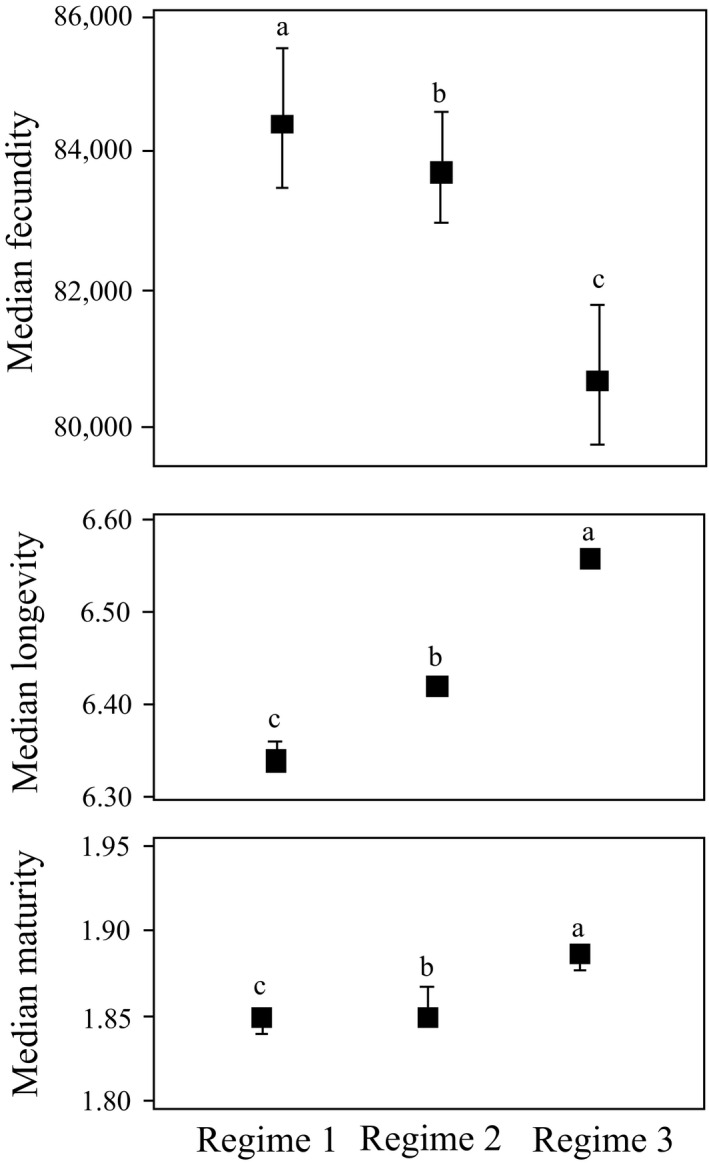
Median of fecundity, longevity, and maturity for the three successional regimes. Pairwise comparison of index differences between the successional regimes was performed with one‐way permutational ANOVA (*p* < .05). For each index, numbers followed by the same letter do not differ significantly

Changes in functional diversity reflected changes in relative abundances of functional groups in each regime. Benthopelagic species increased from 1.2% in regime 1 to 12.9% in regime 3. Lotic species decreased from 83.6% in regime 1 to 63.6% in regime 3, while lentic species increased from 12.3% in regime 1 to 24.6% in regime 3. Fish spawning drift eggs decreased from 89.6% in regime 1 to 75% in regime 3. Long‐distance migratory fish decreased from 59.7% in regime 1 to 5.4% in regime 3. Invertivore fish decreased from 94.3% in regime 1 to 77.6% in regime 3, while piscivore fish increased from 0.6% in regime 1 to 10.8% in regime 3 (Figure 4). CWM of maturity and longevity showed increasing trends along with the three regimes, while CWM of fecundity showed a decreasing trend along with the three regimes (Figure 5). Results of CWM indicated that fish life‐history strategies changed significantly along with the regimes.

Results of the comparison with null models indicated different trait assembly patterns between the three regimes, showing a trend from trait under‐dispersion to trait over‐dispersion (Table 2). FRic, FEve, FDiv, and RaoQ all showed strong evidence of trait under‐dispersion in regime 1. The community in regime 3 displayed clear evidence of over‐dispersion based on FRic and RaoQ. A decrease of functional redundancy was observed, suggesting that species performing similar functions decreased.

**TABLE 2:** Results of one‐tailed test of standard effect sizes (SES) based on the null model for each of the functional diversity indices

	FRic			FEve		FDiv		RaoQ		FR	
	Nb	SES	*p*	SES	*p*	SES	*p*	SES	*p*	SES	*p*
Regime 1	44	**−1.884**	**.027**	**−2.647**	**.002**	**−1.702**	**.044**	**−1.303**	**.044**	1.431	.879
Regime 2	41	0.730	.230	1.033	.843	0.688	.767	−0.513	.444	−0.108	.434
Regime 3	36	**2.495**	**.006**	0.508	.702	0.797	.774	**1.822**	**.041**	**−1.803**	**.002**

Note

Median SES, test statistic, and *p* value are given. Significant results are presented in bold. Negative/positive SES values represent under/over‐dispersion of trait distribution compared to random expectation.

Abbreviations: FDiv, functional divergence; FEve, functional evenness; FR, functional redundancy; FRic, functional richness; Nb, number of species; RaoQ, Rao's quadratic entropy.

## 4 DISCUSSION

The construction of the Three Gorges Dam and the subsequent filling of the Three Gorges Reservoir had significant impacts on both species and functional diversities of fishes in the Yangtze River. The STARS (Figure S1) and MRT (Figure 3) analyses showed the change points of the fish communities in 2004 and 2013, which corresponded to one year following the first filling of the Three Gorges Reservoir in 2003, and 5 years after filling to a 172.5 m water level in 2008, respectively.

Dams and reservoirs are seen as major causes of habitat loss and fragmentation in river ecosystems (Nilsson, Reidy, Dynesius, & Revenga, 2005). The lentic environment upstream of dams forms ecological barriers between river faunal regions (Abell et al., 2008), especially for fish larvae and eggs (Mu, Li, Liu, & Cao, 2014). Dams also obstruct downstream long‐distance migratory fish, preventing them from completing their life history and impacting population survival (Pelicice, Pompeu, & Agostinho, 2015). The upper and middle reaches of the Yangtze River have abundant water with swift currents, and they contain substantial spawning grounds for fishes, especially for those with drift eggs. After the closing and impoundment of TGD in 2003, the longitudinal migration of fish across TGD was blocked (Wu et al., 2004). In addition, most of the fish larvae and drift eggs were stopped in TGR, as the environment had changed to a lake‐like water body. Under these conditions, populations of species at sink localities would go locally extinct without net immigration from source sites (e.g., Pulliam, 1988). Results of SIMPER (Appendix S2: Table S1) and changes of functional groups between regimes (Figure 4) showed migratory fishes (*C. guichenoti*, *R. ventralis*, and *R. cylindricus*) decreased in abundance sharply (45.57%) after the impoundment of TGD. These caused an immediate shift of the downstream fish community after the first impoundment of TGD.

The natural flow regime is considered to be one of the key elements for river ecological integrity and aquatic biodiversity (Lytle & Poff, 2004; Poff et al., 1997). Hydrologic droughts in the Yangtze River at Yichang were aggravated after the operation of TGD began, and flow in October especially decreased sharply (Chen et al., 2016; Li, Dong, et al., 2013; Li, Xiong, et al., 2013), decreasing the maximum discharge while increasing the minimum discharge (Figure 2). Alteration of natural flow can cause the change of functional traits of fish community (Arantes et al., 2019; Pool et al., 2010), such as life‐history strategies (Mims & Olden, 2013), habitat use strategies (Lima et al., 2018), and trophic strategies (Li, Dong, et al., 2013; Li, Xiong, et al., 2013). Our results showed that the relative abundance of fish adapted to a lentic environment increased, while that of fish adapted to a lotic environment decreased (Figure 4). Some fish with new traits, such as *S. chuatsi*, were not present in regime 1, but became more dominant in regime 3. These results are consistent with the idea that community structure was determined by habitat–trait relationships (Villéger et al., 2010). In addition, life‐history traits may play a very important role in the stability of community structure and function (Fujiwara, 2012; Wang, Fujiwara, Gao, & Liu, 2019). Results of CWM (Figure 5) showed that after the operation of TGD, the longevity and maturity of fish in Yichang reach increased, while fecundity decreased significantly. These shifts in life‐history traits may be why the community did not change immediately after the second filling in 2008.

Species diversity reflects only one aspect of the community and may not reveal the true state of the community (McGill, Dornelas, Gotelli, & Magurran, 2015). In this study, compared with regime 2, Shannon–Wiener diversity and Pielou evenness increased in regime 3. However, the fish communities contained less species, lower functional diversity (Table 1), and smaller functional space (Figure 3b). Moreover, species cannot reflect the loss of functional rarity. For example, *C. heterodon* and *C. guichenoti* share very similar ecological traits, except for the ability to migrate. The *C. heterodon* population in Yichang reach can successfully reproduce and recruit after the impoundment of TGD, while the *C. guichenoti* population cannot move across TGD to spawn and, consequently, had an abrupt decrease without recruitment from upstream reaches.

In the present study, the functional diversity of communities has changed among the three regimes. Comparison with the null model revealed that the fish communities in regime 1 were significantly more similar than expected at random, indicating that the fish communities were structured by environmental filtering (Table 2). A similar mechanism was found in tropical fish communities: fish dispersed and selected habitats within expansive flooded areas in wet seasons (Fitzgerald, Winemiller, Sabaj Pérez, & Sousa, 2017). In regime 2, on the other hand, no significant difference from the null model results for all functional diversity indices suggested that the fish communities may have been more stochastically structured. The high functional evenness observed in regime 2 was consistent with the random structure (Table 1). In regime 3, a significantly positive value for Rao's quadratic entropy indicated that the fish communities were more divergent in traits than expected at random, suggesting that the fish communities were structured by limiting similarity. Li et al. (2015) found that species colonization rather than competitive exclusion drives community over‐dispersion. In the present study, we conclude that the cause of trait divergence in regime 3 was consistent with that of Li et al. (2015): the invasion of fish with new traits (mainly *S. chuatsi*) from downstream reaches. The new dominant species *S. chuatsi* is a top predator, which may be playing a role in stabilizing a system that would otherwise be unstable under strong competition.

We emphasize the role of functional diversity in long‐term investigation, and we should pay more attention to functional rarities. Our results indicated that the impacts of dams on downstream fish communities may change over time. Change of maximum and minimum discharge can alter the functional composition of the downstream fish communities. Finally, interactions between species may become more important when the environment is stable.

## CONFLICT OF INTEREST

We declare that we do not have any commercial or associative interest that represents a conflict of interest in connection with the work submitted.

## AUTHOR CONTRIBUTIONS

**Chen Zhang:** Investigation (equal); Methodology (equal); Software (equal); Writing‐original draft (equal); Writing‐review & editing (equal). **Masami Fujiwara:** Methodology (equal); Writing‐original draft (equal); Writing‐review & editing (equal). **Michaela Pawluk:** Methodology (equal); Writing‐review & editing (equal). **Huanzhang Liu:** Conceptualization (equal); Methodology (equal); Supervision (equal). **Wenxuan Cao:** Conceptualization (equal); Methodology (equal); Supervision (equal). **Xin Gao:** Conceptualization (equal); Data curation (equal); Investigation (equal); Methodology (equal); Writing‐review & editing (equal).

## Supporting information

Appendix S1‐S2

## Data Availability

The fish list, discharge datasets, and STARS software are stored in Figshare repository (https://doi.org/10.6084/m9.figshare.12083130).
