# Supplementary material for: Changes in taxonomic and functional diversity of fish communities after catastrophic habitat alteration caused by construction of Three Gorges Dam
Source: Ecol Evol. 2020 Apr 28;10(12):5829–39. doi: 10.1002/ece3.6320 (PMC7319164; doi:10.1002/ece3.6320)
Supplement: Supplementary file 1 — Appendix S1‐S2 [file ECE3-10-5829-s001.docx]

**Appendix S1:** Functional traits used in this study.

**Table S1** Functional traits used to calculated functional diversity indices

| **Functional trait** | **Type of variable** | **Value or range of values** | **Trait modalities for formula** |
| --- | --- | --- | --- |
| Maximum body length | Continuous | 3.2 - 200 | Total length of fishes from fishbase (cm) |
| Vertical position | Categorical | 3 categories | Demersal, benthopelagic, pelagic |
| Rheophily | Categorical | 3 categories | Rheophilic, eurytopic, limnophilic |
| Feeding group | Categorical | 4 categories | Herbivore, invertivore, omnivore, piscivore |
| Mouth position | Categorical | 3 categories | Superior,terminal,inferior |
| Egg type | Categorical | 2 categories | Drifting eggs, demersal eggs |
| Longevity | Continuous | 1 - 13 | Year |
| Age of sexual maturity | Continuous | 0.5 - 4 | Year |
| Fecundity  Migration type | Continuous  Categorical | 127 – 681119  2 categories | Absolute fecundity  Migration,non-migration |
| Body length to caudal-peduncle length ratio | Continuous | 3.5 – 14.5 | Ratio |
| Head length to eye diameter ratio | Continuous | 2.9 – 17.7 | Ratio |
| Body length to body depth ratio | Continuous | 2.3 - 8.4 | Ratio |

**Appendix S2:** Fish species that contributed to the dissimilarity between regimes.

**Table S1** Fish species that contributed to the dissimilarity between regime 1 and regime 2.

| Taxon | Average dissimilarity | Contribution to dissimilarity % | Cumulative contribution % |
| --- | --- | --- | --- |
| *Coreius heterodon* | 22.72 | 38.84 | 38.84 |
| *Coreius guichenoti* | 12.33 | 21.09 | 59.94 |
| *Rhinogobio ventralis* | 6.723 | 11.49 | 71.43 |
| *Rhinogobio cylindricus* | 3.875 | 6.625 | 78.06 |
| *Pelteobagrus vachelli* | 2.714 | 4.64 | 82.7 |
| *Hemiculter leucisculus* | 1.609 | 2.751 | 85.45 |
| *Hemiculter bleekeri* | 1.387 | 2.372 | 87.82 |
| *Saurogobio gymnocheilus* | 1.375 | 2.352 | 90.17 |
| *Pseudolaubuca sinensis* | 0.8017 | 1.371 | 91.54 |
| *Parabramis pekinensis* | 0.5714 | 0.977 | 92.52 |

Notes: Migratory fish are presented in bold italics

**Table S2** Fish species which contributed to the dissimilarity between regime 2 and regime 3

| Taxon | Average dissimilarity | Contribution to dissimilarity % | Cumulative contribution % |
| --- | --- | --- | --- |
| *Coreius heterodon* | 10.85 | 26.77 | 26.77 |
| *Coreius guichenoti* | 5.715 | 14.09 | 40.86 |
| *Siniperca chuatsi* | 4.761 | 11.74 | 52.61 |
| *Pelteobagrus vachelli* | 4.296 | 10.59 | 63.2 |
| *Pseudolaubuca sinensis* | 2.149 | 5.3 | 68.5 |
| *Rhinogobio cylindricus* | 2.067 | 5.097 | 73.6 |
| *Hemiculter leucisculus* | 1.521 | 3.752 | 77.35 |
| *Mystus macropterus* | 1.211 | 2.987 | 80.34 |
| *Xenocypris argentea* | 1.039 | 2.562 | 82.9 |
| *Leiocassis crassilabris* | 1.024 | 2.525 | 85.42 |


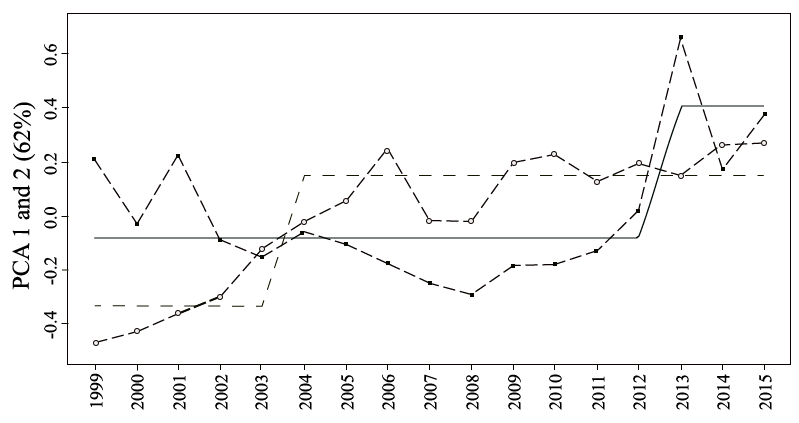


**Figure S1** PCA 1 and 2 of relative abundance are plotted against year. Values of PCA1 and PCA2 are plotted as dashed line with circle dot and squares, respectively. Mean PCA1 and 2 values are plotted as solid line and dashed line, respectively.
